# Effect of various blood glucose levels on regional FDG uptake in the brain

**DOI:** 10.22038/aojnmb.2019.14418

**Published:** 2020

**Authors:** Ismet Sarikaya, Ahmed N. Albatineh, Ali Sarikayaa

**Affiliations:** 1 Department of Nuclear Medicine, Faculty of Medicine, Mubarak Al-Kabeer Hospital, Kuwait University, Kuwait; 2 Department of Community Medicine and Behavioral Sciences, Faculty of Medicine, Kuwait University, Kuwait; 3 Department of Nuclear Medicine, Faculty of Medicine, Trakya University, Edirne, Turkey

**Keywords:** PET, FDG, SUV, Brain, Blood glucose

## Abstract

**Objective(s):**

Studies have mainly assessed the effect of hyperglycemia on ^18^F-fluorodeoxyglucose (FDG) uptake in the brain. In this study, we assessed the FDG uptake of the brain not only in normo- and hyperglycemia but also in hypoglycemia to compare the effect of various blood glucose levels on regional FDG uptake in the brain.

**Methods:**

This retrospective study was conducted on whole-body FDG positron emission tomography/computed tomography (PET/CT) images including the brain. The inclusion criteria included adult patients with no known history of diseases or symptoms affecting the brain, lack of abnormal brain findings on both PET and CT images, no image artifacts, and lack of any factors affecting brain FDG uptake. Maximum standardized uptake values (SUV_max_) were measured in the lateral and medial frontal, temporal, parietal, and occipital cortices, lateral cerebellar cortex, posterior cingulate cortex, caudate nucleus, putamen, thalamus, brain stem (BS), and scalp in patients with normal (91-100 mg/dl), low (61-70 mg/dl), and high (171-200 mg/dl) blood glucose (BG) levels. Mean SUV_max_ of the brain regions for each BG range was calculated and statistically analyzed.

**Results:**

In all BG levels, FDG uptake was at the highest level in the lateral frontal cortex and lowest level in the medial temporal cortex (MTC) and BS. The SUV_max_ in all assessed brain regions was significantly lower in hyperglycemia (P<0.001). However, this value was not significantly different in hypoglycemia (P>0.05) as compared to that in normoglycemia. At the BG range of 171-200 mg/dl, hyperglycemia-induced reduction in regional SUV_max _had a range of 55.9-63.7% (60%±2.4%). This reduction was below 60% in the MTC, cerebellum, and BS and above 60% in other regions. Scalp activity was lower in hyperglycemia (P<0.001) and not different in hypoglycemia (P>0.05) as compared to normoglycemia.

**Conclusion:**

The FDG uptake appears to be at the highest level in the lateral frontal cortex and the lowest level in the MTC and BS in normo-, hypo-, and hyperglycemia. Hyperglycemia-induced reduction in FDG uptake was approximately the same as that in various regions of the brain. However, the MTC, cerebellum, and BS may be slightly less affected than the other regions. Hypoglycemia does not seem to have a significant effect on FDG uptake in the brain.

## Introduction

 Glucose is the main source of energy for the brain. The brain consumes 20% of total body glucose-derived energy in humans. Both neurons and astrocytes are the main consumers of glucose (1-3). Glucose-derived energy through the generation 

of adenosine triphosphate (ATP) is used for physiological brain functions, neuronal and non-neuronal cellular maintenance, and generation of neurotransmitters (1). The large blood-to-brain concentration gradient drives the facilitative transport of glucose across the endothelial membranes via glucose transporter-1 (GLUT-1) into extracellular fluid (1). The GLUT-1 further mediates glucose uptake from extracellular fluid into astrocytes, oligodendroglia, and microglia, whereas GLUT-3 facilitates neuronal glucose uptake (1, 2). The GLUT-3 has a much higher transport rate than GLUT-1. 

 Intracellular glucose is phosphorylated by hexokinase I to glucose-6-phosphate. Glucose-6-phosphate is metabolized via the glycolytic pathway to generate ATP. Glucose-6-phosphate is also used for the pentose phosphate shunt pathway to generate reduced nicotinamide adenine dinucleotide phosphate to manage oxidative stress and synthesize nucleic acid precursors (1). In astrocytes, glucose-6-phosphate is the precursor for glycogen, which is the brain’s only energy reserve. 

 Positron emission tomography (PET) imaging of the brain with radiolabeled glucose analog, namely ^18^F-fluorodeoxyglucose (^18^F-FDG or FDG), is a commonly used study for the early and differential diagnosis of dementia. Brain FDG PET imaging is also used in cases with primary or metastatic brain tumors and to a lesser extent, in various other neurological and psychiatric conditions. The FDG uptake in the brain is similar to glucose uptake with the exception of proceeding to glycolysis. Once taken up by the cells via glucose transporters, ^18^F-FDG is phosphorylated into FDG-6-phosphate by hexokinase (glucokinase) enzyme. The FDG-6-phosphate is mainly trapped in the cells as it minimally undergoes subsequent metabolism and has a low dephosphorylation rate. 

 The PET studies have demonstrated that high blood glucose reduces FDG uptake in the brain (4-9). In this regard, high blood glucose competes with FDG and reduces FDG uptake in the normal brain. In addition, in the presence of high blood glucose, endogenous insulin level increases which causes higher FDG uptake in insulin-sensitive normal tissues (adipose tissue and muscle via GLUT-4), thereby further reducing FDG uptake in the brain. The FDG uptake in the brain is inversely related to blood glucose level; in this respect, a higher blood glucose level is associated with lower FDG uptake in the brain (4, 9).

 As the brain FDG uptake is closely affected by blood glucose level, the metabolic activity of the brain can be more accurately assessed if FDG is injected when blood glucose is within normal or near normal limits (90-100 mg/dl ±10). However, in routine studies, it is not always possible to obtain images at an optimal blood glucose level. Accordingly, guidelines recommend obtaining FDG PET brain images when blood glucose is lower than 160 or 150-200 mg/dl (10, 11).

 Previous studies have mostly assessed the global and regional FDG uptake in the brain in normo- and hyperglycemic conditions. Regarding this, the present study was conducted to assess the FDG uptake of the brain not only in normo- and hyperglycemic conditions but also in hypoglycemic state to compare the effect of various blood glucose levels on regional FDG uptake in the brain.

## Methods

 In this retrospective study, whole-body FDG PET/computed tomography (CT) images of adult patients were selected for further analysis. This retrospective study was approved by the Ethics Committee of the Kuwait Ministry of Health. The FDG PET/CT images were obtained at the Mubarak Al-Kabeer Hospital, Kuwait. Whole-body FDG PET/CT images were obtained mainly for oncology patients. The PET/CT images were obtained at the Philips Gemini Time of Flight PET/CT camera (Philips Medical Systems, Best, Netherlands). These images were acquired 60 min following the intravenous injection of 222 MBq (6 mCi) ^18^F-FDG. 

 Blood glucose levels were measured using a glucometer before injecting ^18^F-FDG. At our institute, ^18^F-FDG is injected when the blood glucose is ≤ 150 mg/dl. However, the health team sometimes proceeds with ^18^F-FDG injection although blood glucose is higher. Prior to PET image acquisition, a low-dose CT was obtained for attenuation correction and anatomic localization purposes. The PET images were acquired for 2-3 min/bed position from the top of the head to mid-thighs or toes. The images were corrected for attenuation on the basis of the CT data, reconstructed using a standard iterative algorithm, and reformatted into transaxial, coronal, and sagittal views. Maximum intensity projection images were also generated. Both attenuation-corrected and -uncorrected PET images, as well as PET/CT fusion images, were reviewed. 

 The PET/CT images of adult patients within the age range of 18-65 years with no known history of brain disorders or symptoms, as well as no visually abnormal findings on PET and low-dose CT images, were selected for further analysis. On the other hand, the images affected by any of the following situations were excluded: high amount of extravasated activity, motion artifacts in the head region, and extracranial intense uptake in large lesions which can cause a competitive decrease in FDG uptake in the brain. Patients younger than 18 years and older than 65 years were excluded from the study due to age-related differences and changes in regional brain metabolism. 

 We measured maximum standardized uptake value (SUV_max_) in the various regions of the brain in patients with the following blood glucose ranges: normal blood glucose (91-100 mg/dl), low blood glucose (61-70 mg/dl), and high blood glucose (171-200 mg/dl). In the hyperglycemic group, we decided to select cases with a blood glucose level of 171-200 mg/dl because we wanted to see the maximum possible effect of hyperglycemia on brain FDG uptake. We did not have enough patients with blood glucose exceeding 200 mg/dl. In each glucose range, fifteen FDG PET/CT images were analyzed (i.e, a total of 45 patients and images). 

 Regions of interests (ROIs) were placed in the lateral and medial cortices of the frontal, temporal, parietal, and occipital lobes, lateral cerebellar cortex, posterior cingulate cortex, caudate nucleus, putamen, thalamus, and brain stem to measure SUV_max_. The SUV_max_ of the scalp was also measured. The ROIs were circular with the diameters of 0.5-1 cm depending on the area selected. 

 The data management and statistical analysis were carried out using the SPSS software, version 25.0 (IBM Corp.). The SUV_max_ of brain regions and scalp were presented as mean±standard deviation. Since the distribution of brain activity was not normally distributed, and the variances across brain glucose levels were not homogeneous, ANOVA could not be implemented. Therefore, the nonparametric test of Kruskal-Wallis test was run to compare the distribution of brain activity across three brain glucose levels for several brain regions. In addition, to compare SUV_max_ for two brain glucose levels, the non-parametric Mann-Whitney U test was implemented. All tests were two-tailed, and the probability value of < 0.05 was considered statistically significant.

## Results

**Figure 1:**
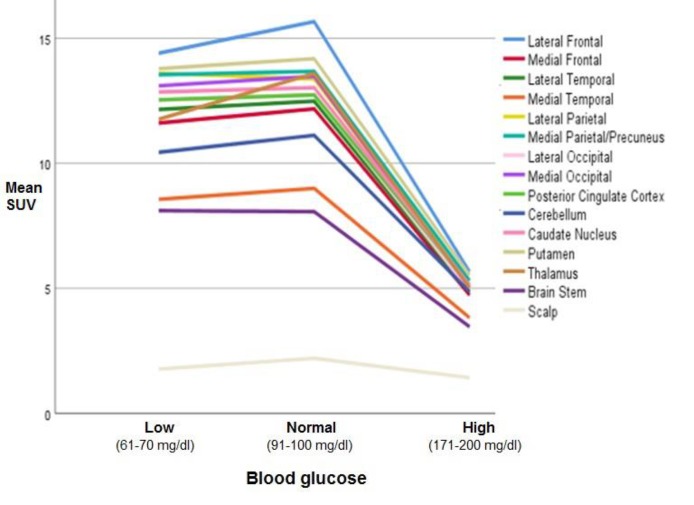
Mean regional SUV_max_ in normal, low, and high blood glucose levels

**Figure 2:**
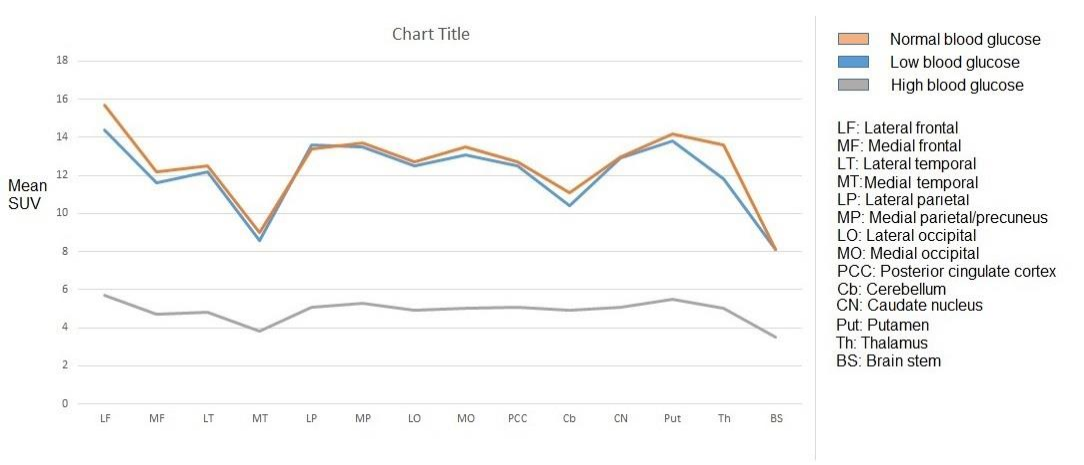
Mean regional SUV_max_ in normal, low, and high blood glucose levels

 We assessed images of 45 adult patients (i.e, 23 females, and 22 males) with a mean age of 50.1±12.3 years (range: 21-65 years). The mean SUV_max_ of all assessed brain regions in normoglycemia, hyperglycemia, and hypoglycemia are shown in Figures 1 and 2. The mean regional SUVmax had the ranges of 8.1±1.3 to 15.7±2.9, 8.1±1.6 to 14.4±4.2, and 3.5±0.8 to 5.7±1.1 in normoglycemia, hypoglycemia, and hyperglycemia, respectively. In all blood glucose levels, FDG uptake was at the highest level in the lateral frontal cortex and the lowest level in the medial temporal cortex and brain stem. 

 In both normo-and hypoglycemia, mean SUV_max_ was below 10 in the medial temporal cortex and brain stem and above 10 in other brain regions. In addition, in hyperglycemia, mean SUV_max_ was below 4 in the medial temporal cortex and brain stem and above 4 in other regions. There was no significant difference between the SUV_max_ of hypoglycemia and normoglycemia in all brain regions (P>0.05).

**Figure 3:**
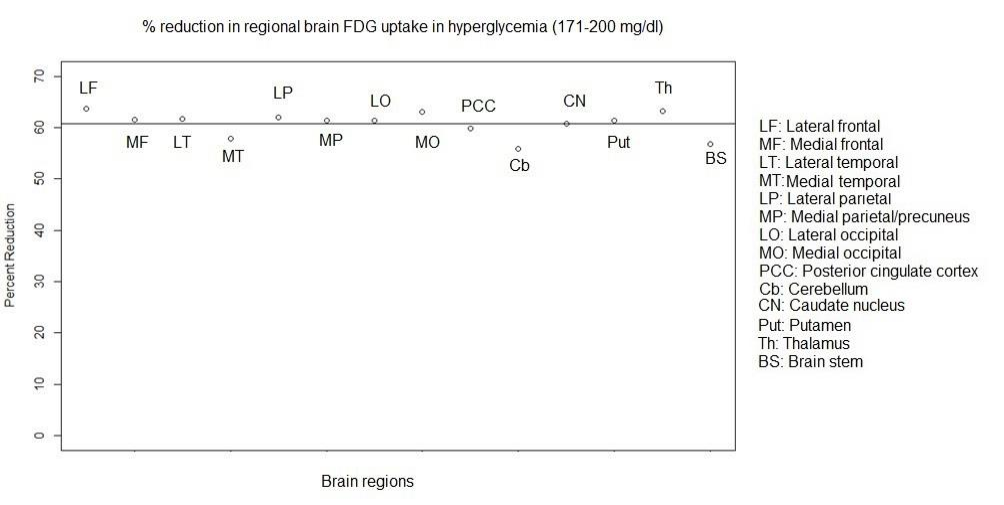
Percent regional reduction in FDG uptake/SUV_max _in hyperglycemia at a blood glucose level of 171-200 mg/dl (Horizontal line represents mean percentage reduction.)

 In hyperglycemia, at a blood glucose level of 171-200 mg/dl, the mean SUV_max_ of all the assessed brain regions was significantly lower as compared to those in normoglycemia (P<0.001). Mean percent regional reduction in SUV_max_ was 60.7±2.4% (range: 55.9% to 63.7%). In addition, the percent regional decreases in SUV_max_ were obtained as 63.7%, 61.5%, 61.6%, 57.8%, 61.9%, 61.3%, 61.4%, and 63%,in the lateral frontal, medial frontal, lateral temporal, medial temporal, lateral parietal, medial parietal/precuneus, lateral occipital and medial occipital cortices, respectively. These rates were estimated at 59.8%, 55.9%, 60.8%, 61.3%, 63.2%, and 56.8%, in the posterior cingulate cortex, cerebellar cortex, caudate nucleus, putamen, thalamus, and brain stem, respectively (Figure 3). 

Mean SUV_max_ of the scalp was obtained as 2.2±0.4, 1.8±0.6, and 1.4±0.3 in normoglycemia, hypoglycemia, and hyperglycemia, respectively. Mean SUV_max_ of the scalp was significantly lower in hyperglycemia (P<0.001) than those of the two other groups. However, this value was not significantly different between hypoglycemia (P>0.05) and normoglycemia groups. In hyperglycemia, percent reduction of SUV_max_ in the scalp was 36.4% as compared to approximately 60% reduction in the brain.

## Discussion

 Various studies have analyzed FDG uptake in the brain, either globally, or in one or multiple regions, mainly in normoglycemic and hyperglycemic conditions. To assess FDG uptake in the brain, studies either used a dedicated brain PET image or whole-body FDG PET images of oncologic patients. Based on the evidence, hyperglycemia reduces FDG uptake in the brain (4-9). Reduction of FDG uptake in the brain starts after a blood glucose level of 110 mg/dl and gradually becomes more prominent with increasing blood glucose levels (4, 9). In our recent study, targeted toward the measurement of SUV_max_ in the frontal cortex in whole-body images, there were reduction rates of approximately 20%, 35%, 50%, 60%, and 65% from the normal level at the blood glucose levels of 111-120, 121-140, 141-160, 161-200, and ≥ 201 mg/dl, respectively (9). 

 Healthy subjects show variants of FDG distribution depending on their age or gender (12). The FDG uptake is usually homogeneous and symmetrical; however, the areas of slightly higher activity are observed in the basal ganglia, frontal eye fields, posterior cingulate cortex, and visual cortex (12). This activity is relatively lower in the medial temporal cortex (12). In the current study, there were regional differences in FDG uptake in all blood glucose levels. The FDG uptake/SUV_max_ was at the lowest level in the brain stem and medial temporal cortex and highest level in the lateral frontal cortex, followed by the putamen, in all blood glucose levels. 

 In a study conducted on healthy adults, the areas with the highest glucose metabolisms were the posterior cingulate gyri, thalami, basal ganglia, and visual cortex (13). In the mentioned study, the lowest values were found in the occipital cortex and cerebellum (13). In females, a lower uptake was reported in the cerebellum as compared to that in the supratentorial regions (14). In our study, cerebellar FDG uptake was lower than that of frontal, parietal, occipital, and lateral temporal cortices and basal ganglions. Regional differences in females and males were also reported (15). 

 Studies have assessed global or regional glucose metabolism in the brain in hyperglycemia. While some studies reported that there is a more significant reduction in glucose metabolism in Alzheimer’s disease (AD)-related regions, others did not find more significant changes in specific regions (16-21). Kawasaki et al., obtaining dynamic PET scans in fasting and mild hyperglycemic conditions from healthy subjects (mean age of 66.2 years), found that in the mild hyperglycemic condition, FDG uptake relatively decreased in the gray matter (16). This reduction was mainly in the frontal, temporal, and parietal association cortices, posterior cingulate, and precuneus in both statistical parametric mapping of global brain and the gray and white matter regions computed based on magnetic resonance image reference-based analyses (16). When the cerebellar cortex was adopted as the reference region, those decreasing patterns disappeared in their study. In cognitively normal older patients (74.4±1.4 years) with prediabetes or early type II diabetes, greater insulin resistance was associated with a reduced uptake of FDG in AD-related regions in the frontal, temporal-parietal, and cingulate regions (17). 

 Burns et al. reported that higher fasting serum glucose levels were significantly correlated with lower regional cerebral metabolic rate for glucose and confined to the vicinity of brain regions preferentially affected by AD (temporal, bilaterally in the precuneus/posterior cingulate, parietal, prefrontal, and occipital) in patients with a first-degree family history of AD with a similar pattern in APOEε4 noncarriers and carriers (18). Focusing on AD-related regions, Ishibashi et al. found a significantly lower uptake of FDG and O-15 water in the precuneus/posterior cingulate, lateral parietal cortex, and frontal cortex after glucose loading (19). In a longitudinal study conducted on four cognitively normal elderly subjects with diabetes, the distribution pattern of FDG changed depending on plasma glucose levels in an individual (20). In the mentioned study, the AD-like pattern could appear or disappear with increasing or decreasing plasma glucose levels, respectively (20). 

 In a recent study, Viglianti et al. investigated the use of whole-body FDG PET images of oncologic patients and semiquantitative analysis of the brain (21). They found no regional hyperglycemic effect (blood glucose of 128.4±5.6 mg/dl) on FDG uptake in the brain when subjects were normalized using pons or cerebellum, but regional hyperglycemic effects were seen when normalizing by the whole brain (21). 

 In the current study, the hyperglycemia-induced percent reduction in brain FDG uptake was approximately the same degree in the various regions of the brain, and in particular, it was not more significant in AD-related regions (i.e., the medial parietal/precuneus, lateral parietal, lateral temporal, and posterior cingulate cortex). In the study carried out by Burns et al., all the patients had a first-degree family history of AD with or without APOEε4 carriers which may be the reason for finding abnormal findings in AD-related regions (18). 

 In our study, hyperglycemia was chronic or stress-related as all our patients fasted 6 h before the implementation of the PET scan. In a study performed by Ishibashi, hyperglycemia was acute (glucose load) (19). Acute and chronic hyperglycemia may have different effects on FDG uptake in the brain which may also show regional differences (5, 22). In a couple of studies carried out by Baker et al. and Burns et al., fasting hyperglycemia was reported to be at a mild level (107 mg/dl and maximum of 126 mg/dl, respectively) (17, 18). Ishibashi investigating glucose loading reported a mean blood glucose level of 122.3±22.2 mg/dl (19). In our study, the blood glucose level was significantly higher (171-200 mg/dl) than the level presented in the above-mentioned studies. 

 Regional differences in brain FDG uptake may be affected by the level of hyperglycemia. In hyperglycemia, we found a significant reduction in FDG uptake in all the assessed brain regions as compared to that in normoglycemia. Hyperglycemia induced a reduction of approximately 60% in SUV_max_ at the blood glucose range of 171-200 mg/dl. In the brain stem, medial temporal cortex, and cerebellar cortex, the percent reduction was slightly lower as compared to that in other regions. However, the significance of this reduction was uncertain due to the gross/approximate measurement of FDG uptake in the brain in our study. 

 In the current study, there was no significant difference in the mean SUV_max_ of the regions in hypoglycemia as compared to that in normoglycemia. Hypoglycemia may activate the compensatory mechanisms of glucose metabolism in the brain (23, 24). Cerebral blood flow and glucose delivery are increased at a plasma glucose level of < 2 mmol/L in acute hypoglycemia (24). 

In the present study, the SUV_max_ of the scalp was lower in hyperglycemia as compared to that in normoglycemia. However, the FDG uptake was affected/reduced in a lower degree in the scalp as compared to that in the brain in hyperglycemia. This can make the brain/scalp SUV ratio unhelpful when comparing two studies of the same patient with different blood glucose levels. However, the measurement of SUV_max_ in the scalp may help to differentiate hyperglycemia-related diffusely reduced uptake from disease-related uptake reduction in the brain. 

 One of the limitations of our study is the gross assessment of regional brain FDG uptake using the brain images of whole-body PET/CT scan (2-3 min acquisition per bed, and usually in the arms up position) rather than utilizing dedicated images of the brain (10-15 min acquisition in arms down position). In the gross analysis of regional brain FDG uptake using whole-body images, the regional differences of <10% may not be significant. However, when using dedicated brain PET images or particularly dynamic PET images with arterial blood sampling at multiple time points for the absolute quantification of regional metabolic rates, regional differences lower than 10% may be significant. 

 In addition, in our study, we assessed only 14 major brain regions. The brain has 52 Brodmann areas in each hemisphere, and each area has a unique function. Multimodal MR images delineated 180 areas per hemisphere bounded by sharp changes in the cortical architecture, function, connectivity, or topography (25). Assessment of regional glucose metabolism in small or specific brain regions requires accurate methods to localize those regions, such as patients’ own PET/MR images. Evaluation of posterior cingulate cortex activity in our study was limited due to the poor resolution and low count of images and small size of this area. In some other studies, either dedicated brain PET or dynamic PET with or without MR correlation and also various semi-quantification programs were used. 

 In the current study, we were unable to use our available semi-quantitative brain analysis programs because our brain images were part of whole-body images, rather than dedicated brain images. However, semi-quantitative analysis programs should also be used carefully as they have certain limitations (26). Another limitation of our study was the non-evaluation of the effect of chemotherapy on brain metabolism in patients with malignancies. Patients who undergo systemic chemotherapy develop a reduction in their brain metabolism due to the phenomenon known as “Chemo-Brain” (27, 28).

## Conclusion

 The FDG uptake appeared to be at the highest level in the lateral frontal cortex and the lowest level in the medial temporal cortex and brain stem in all blood glucose levels. Hyperglycemia-induced reduction in FDG uptake was approximately at the same degree in the various regions of the brain. However, the medial temporal cortex, cerebellum, and brain stem might be slightly less affected than the other regions of the brain. Hypoglycemia did not seem to have a significant effect on FDG uptake in the brain.

## Disclosure

 No potential conflict of interest relevant to this article was reported.
